# Maternal–Fetal Exposure to Oncoelements and Their Oxidative and Epigenetic Impact on Pregnancy Outcomes

**DOI:** 10.3390/ijms27020669

**Published:** 2026-01-09

**Authors:** Joanna Grzesik-Gąsior, Agnieszka Bień, Katarzyna Zalewska, Michał Nieszporek, Katarzyna Witkowska, Anna Merklinger-Gruchała

**Affiliations:** 1 State University of Applied Sciences in Krosno, Rynek 1, 38-400 Krosno, Poland; katarzyna.zalewska@pans.krosno.pl; 2 Chair of Obstetrics Development, Faculty of Health Sciences, Medical University of Lublin, 20-081 Lublin, Poland; agnieszka.bien@umlub.edu.pl; 3 Endoscopic Simulation Centre, Centre of Postgraduate Medical Education, 01-813 Warsaw, Poland; michal.nieszporek@cmkp.edu.pl; 4 Institute of Nursing, Department of Integrated Medical Care, Faculty of Health Sciences, Collegium Medicum, The Masovian University in Plock, 09-402 Plock, Poland; k.witkowska@mazowiecka.edu.pl; 5 Faculty of Health Sciences, Medical College, Andrzej Frycz Modrzewski Krakow University, 30-705 Krakow, Poland; amerklinger-gruchala@uafm.edu.pl

**Keywords:** trace elements, pregnancy, placenta, cord blood, heavy metals, oxidative stress, prenatal exposure, delayed effects

## Abstract

The proper course of pregnancy and fetal development depends, among other factors, on maintaining adequate levels of micronutrients in the maternal body. This integrative, concept-driven narrative review summarizes the current state of knowledge on the impact of selected elements, referred to as oncoelements, on placental function and obstetric outcomes. These include both potentially protective elements (selenium, zinc, copper) and toxic metals (cadmium, lead, arsenic), which, in excess may disrupt oxidative, hormonal, and epigenetic homeostasis. Rather than providing a quantitative synthesis, the article is structured around a four-level conceptual model integrating molecular mechanisms, placental protection, clinical outcomes, and umbilical cord blood as a biomarker of prenatal exposure. Mechanisms of toxicity include oxidative stress, mitochondrial dysfunction, DNA damage, and altered gene expression. Given the observational nature of most studies, clinical recommendations remain cautious. Micronutrient assessment may be useful in selected high-risk groups, but requires further validation. In environmentally burdened regions, screening for toxic metals may be considered. Future research should clarify dose–response relationships, define threshold concentrations, and explore molecular biomarkers of exposure. Umbilical cord blood offers a promising matrix for assessing fetal exposure, although interpretation is limited by methodological variability and the lack of reference values.

## 1. Introduction

The proper course of pregnancy and fetal development is strongly dependent on the balance of micronutrients in the maternal body. Essential trace elements perform regulatory functions in numerous biological processes, including redox homeostasis, endocrine regulation, epigenetic programming, and mitochondrial function. The accumulation of heavy metals, as well as micro- and macroelements, begins as early as the fetal stage. Early prenatal exposure to such elements, particularly toxic metal(loid)s, may have delayed effects only after a certain latency period. Therefore, their impact can be observed not only during pregnancy and childbirth but also in the neonatal period (e.g., reduced birth weight or length) and later stages of development, when neurological, behavioral, or metabolic disorders may emerge [1,2].

Assessing the impact of environmental pollutants on human health is challenging due to various confounding factors, such as population mobility, dietary differences, and interactions between chemical compounds, for example, components of tobacco smoke. Umbilical cord blood, collected at delivery, is increasingly utilized in prenatal research as a non-invasive and sensitive biomarker of fetal exposure to toxic substances. It reflects the actual level of exposure to trace elements and heavy metals during pregnancy. Therefore, such elements should be analyzed not only individually but also as part of complex chemical mixtures, which may interact in terms of bioavailability and toxicity [2].

The co-occurrence of various substances may modify the individual effects of each component. Measuring individual elements may thus be insufficient to fully assess fetal exposure. Both individually and in mixtures, inorganic elements have been associated with reduced anthropometric parameters in newborns [3,4,5].

Trace elements play an essential role in regulating biochemical processes. While some are vital for maintaining homeostasis, others, especially when present in excess, can reach toxic concentrations and disrupt physiological functions. These metals accumulate in body tissues; some can be stored and later utilized in metabolic processes, whereas others require chelation for elimination. Imbalances may arise not only from overexposure but also due to nutritional deficiencies or drug interactions [6,7].

In this review, we use the term oncoelements to describe a group of trace elements whose concentrations, whether deficient or excessive, may influence key molecular pathways such as oxidative stress regulation, apoptosis, DNA repair, and epigenetic programming. This category includes both essential elements with protective potential (for example, selenium, zinc, and copper) and toxic elements (such as cadmium, lead, and arsenic). Although the term is not widely standardized in the literature, it is used here as a unifying conceptual label, partly overlapping with descriptors such as “trace elements with redox-modulating and carcinogenic potential.” Importantly, we do not imply a direct causal relationship with cancer. Rather, we focus on mechanistic and associative evidence relevant to prenatal development within the framework of the Developmental Origins of Health and Disease (DOHaD) [6,8].

## 2. Selection Criteria and Conceptual Framework

### 2.1. Review Design and Search Strategy

This paper is an integrative, concept-driven narrative review that develops a four-level conceptual model linking: molecular and cellular mechanisms of trace element action, the placenta’s protective and regulatory functions, pregnancy and neonatal outcomes, and umbilical cord blood as a biomarker of prenatal exposure. Rather than performing a quantitative meta-analysis, this approach enables the integration of heterogeneous evidence derived from observational clinical research, and biomarker-based investigations, which differ substantially in design, exposure assessment, and outcome definitions. Only studies conducted in human populations were included; animal and in vitro studies were intentionally excluded to maintain clinical and translational relevance.

The literature search was conducted using PubMed/MEDLINE, Embase, and Scopus, and was supplemented by manual screening of reference lists from relevant articles. The search covered publications from the last decade, was limited to studies published in English and focused on experimental, clinical, and epidemiological studies selected for their conceptual relevance to placental function, pregnancy and neonatal outcomes, and biomarkers of prenatal exposure. Given the narrative design, the review is susceptible to selection bias. Moreover, no formal risk-of-bias tool was applied, which represents an inherent methodological limitation.

The overall strength and consistency of evidence for individual oncoelements were categorized qualitatively using principles informed by the GRADE framework. Evidence was classified as limited, moderate, or consistent based on study design, coherence across findings, and biological plausibility, without numerical scoring [9].

### 2.2. Conceptual Foundation

This review is grounded in the Developmental Origins of Health and Disease (DOHaD) paradigm, which posits that environmental exposures during critical periods of intrauterine development can shape long-term health trajectories. The elements analyzed in this review were selected based on their relevance to placental function and pregnancy outcomes, particularly in relation to redox imbalance, epigenetic modulation, and toxicological effects consistent with the DOHaD paradigm.

The inclusion criteria for elements discussed in this review were:

1. Well-established involvement in redox homeostasis and enzymatic antioxidant defense;
2. Evidence of genotoxic potential, particularly for elements capable of inducing oxidative DNA damage or altering epigenetic regulation;
3. Documented epidemiological associations with pregnancy complications or altered neonatal anthropometric outcomes;
4. Potential for biological antagonism or synergism within the maternal–fetal mineral balance (e.g., Zn/Cd, Se/Cu interactions).

Based on these criteria, six key oncoelements were identified as most relevant to maternal–fetal health: selenium (Se), zinc (Zn), and copper (Cu) as essential trace elements (deficiency- and excess-sensitive) involved in antioxidant and enzymatic defense, and arsenic (As), cadmium (Cd), and lead (Pb) as toxic elements with pro-oxidative and epigenetically disruptive properties. Selenium, zinc, and copper support proper placental function, regulation of apoptosis, and fetal growth by maintaining oxidative balance and stabilizing DNA repair processes. In contrast, arsenic, cadmium, and lead interfere with enzymatic systems, disrupt mitochondrial activity, and alter DNA methylation, leading to increased oxidative stress, impaired nutrient transport, and restricted intrauterine growth [7,8,10].

This dualistic framework distinguishes essential and toxic oncoelements and allows a comprehensive molecular-level interpretation of how trace element imbalances may contribute not only to carcinogenic pathways but also to pregnancy pathophysiology. Understanding their interplay offers insight into shared molecular mechanisms linking environmental exposure, oxidative stress, and the developmental origins of health and disease.

The identified maternal–fetal model, which combines sources of environmental exposure, placental biology, molecular mediators, and observable clinical outcomes, along with synchronized measurement streams (maternal blood, placenta, umbilical cord blood) have been structured within the DOHaD lens. We propose a four-level conceptual framework:

Level 1—Biological role of elements in the body and molecular mechanisms. Describes how each oncoelement affects oxidative stress, apoptosis, DNA repair, and epigenetic programming.

Level 2—Protective functions of the placenta in the context of maternal oncoelement exposure. This level explores the role of the placenta as both a protective filter and a site of element accumulation or dysfunction.

Level 3—Pregnancy and newborn outcomes. Summarizes evidence linking element exposure to fetal growth restriction, low birth weight, preterm birth, and other outcomes.

Level 4—The role of umbilical cord blood as a biomarker. Highlights umbilical cord blood and placental tissue as accessible biomarkers that reflect both maternal exposure and fetal physiological status.

In the context of the DOHaD framework, trace element exposure during critical windows of fetal development may exert long-lasting effects on offspring health that extend well beyond birth outcomes. A growing body of evidence suggests associations with neurodevelopmental, cardiometabolic, and reproductive consequences manifesting in later life. Prenatal exposure to toxic elements such as lead, arsenic, or cadmium has been linked to impaired cognitive development, behavioral problems, and altered brain structure during childhood and adolescence [11,12]. In parallel, dysregulation of essential trace elements including zinc and selenium may interfere with neurogenesis and synaptic plasticity, increasing susceptibility to neurodevelopmental disorders. From a cardiometabolic perspective, early-life exposure to toxic metals or micronutrient imbalances may disrupt endocrine and metabolic programming, thereby increasing the risk of obesity, hypertension, and insulin resistance later in life [8,13]. These outcomes are thought to be mediated by oxidative stress, mitochondrial dysfunction, and epigenetic reprogramming of metabolic pathways. Emerging evidence also suggests long-term impacts on reproductive health, including altered pubertal timing, hormonal imbalances, and reduced fertility. For example, prenatal exposure to endocrine-disrupting elements such as arsenic, lead, or mercury may interfere with gonadal development and the regulation of the hypothalamic–pituitary–gonadal axis, with potential consequences for reproductive capacity in adulthood [12,13,14]. Collectively, these findings underscore the importance of considering lifespan health trajectories when evaluating the impact of prenatal trace element exposure, and further support the use of the DOHaD framework as a guiding model in environmental, developmental, and reproductive epidemiology.

Trace element exposure varies widely across geographic regions due to environmental, dietary, and industrial differences. Populations in areas affected by mining, groundwater contamination, or traditional diets may experience elevated exposure to toxic metals such as arsenic, cadmium, and lead, while essential element deficiencies (e.g., selenium, zinc) may be more common in regions with poor soil or limited dietary diversity. For example, studies from Japan and Inuit populations highlight distinct exposure profiles and fetal outcome associations linked to regional factors [15,16]. Likewise, arsenic exposure remains a major public health concern in certain parts of the world, with implications for maternal and fetal health [17]. These geographic differences limit the direct generalizability of findings and underscore the need for region-specific risk assessments and exposure mitigation strategies.

Accumulating evidence suggests that the effects of prenatal exposure to trace elements may differ by fetal sex, reflecting sexually dimorphic responses in placental function, oxidative stress regulation, and epigenetic programming. Several studies report that male fetuses may be more vulnerable to adverse outcomes such as fetal growth restriction and neurodevelopmental deficits following exposure to toxic metals like cadmium or lead, possibly due to lower antioxidant capacity and differential gene expression profiles [11,18]. Conversely, some data suggest heightened susceptibility in females to endocrine-disrupting effects, particularly in relation to pubertal development and reproductive outcomes [13,14]. These differences may arise from sex-specific placental adaptations, including differences in nutrient transport efficiency, mitochondrial function, and hormone metabolism. Recognition of such dimorphic responses is essential for refining risk assessment models and advancing precision prevention strategies in maternal–fetal health research.

**Figure 1:**
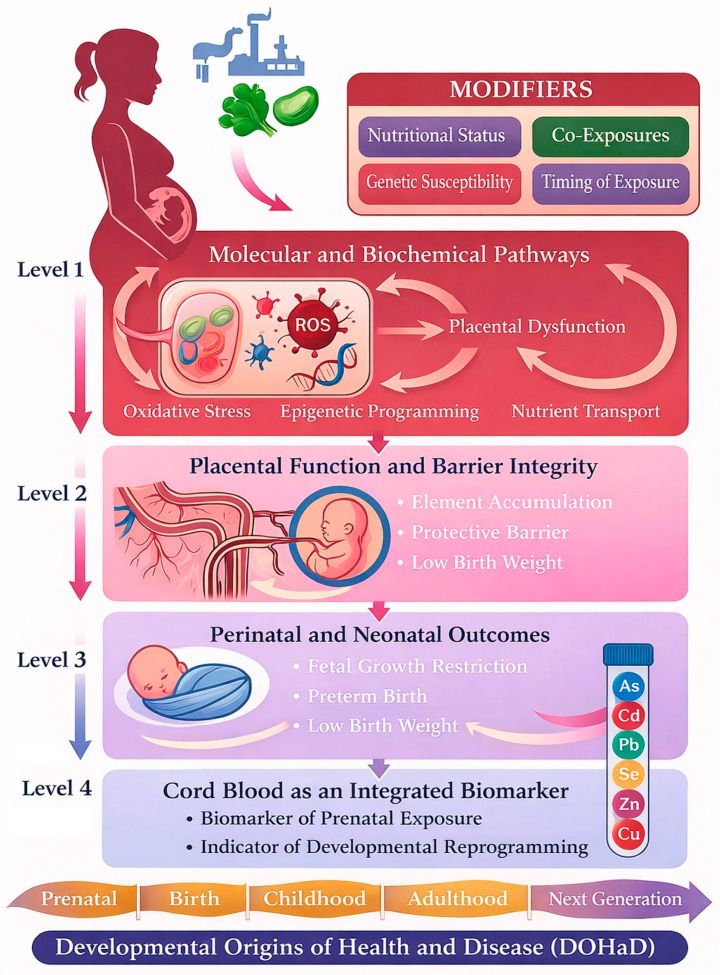
Conceptual model illustrating how maternal exposure to oncoelements may affect fetal development and pregnancy outcomes within the DOHaD framework.

While prior reviews typically summarize trace element toxicology either at the mechanistic level or at the level of pregnancy outcomes, our four-level model explicitly links molecular nodes (redox, mitochondrial, endocrine, epigenetic), the placenta as a biological mediator and selective barrier with regulated transporter expression, perinatal and neonatal endpoints, and synchronized biomarker matrices (maternal blood–placenta–cord blood) as measurable “readouts” of fetal exposure. The model also integrates mixture effects and antagonistic/synergistic mineral balance (e.g., Zn/Cd, Se-dependent antioxidant systems) as modifiers of pathway activation, consistent with the DOHaD perspective. Finally, by mapping outcomes to exposure matrices and timing, the framework supports hypothesis generation regarding critical windows of susceptibility and improves comparability across heterogeneous studies. Core DOHaD mechanisms are illustrated in Figure 1, which integrates molecular perturbations, placental mediation, perinatal outcomes, and potential life-course trajectories.

## 3. The Biological Role of Elements in the Body and Molecular Mechanisms

At the molecular level, both essential and toxic elements influence pregnancy through complex, interrelated biochemical and epigenetic pathways that regulate oxidative stress, apoptosis, and gene expression in maternal and placental tissues. The formation of reactive oxygen species (ROS) is a natural part of cellular metabolism, but becomes harmful when antioxidant mechanisms are insufficient. Importantly, the strength of evidence differs across mechanisms and elements. Oxidative stress and disrupted placental redox balance are consistently supported across multiple human studies and mechanistic data, whereas endocrine-disrupting and long-term programming claims are more heterogeneous and, in parts, based on emerging evidence. To improve interpretability, we distinguish between well-established pathways, emerging associations, and hypothesis-generating links throughout this section.

Oncoelements modulate gene expression through epigenetic modifications such as DNA methylation, histone modifications, and microRNA regulation. Arsenic and cadmium are well-documented modulators of the epigenome. They induce global DNA hypomethylation and local hypermethylation of genes controlling oxidative response, growth factors, and cell cycle (e.g., GSTP1, MT1, IGF2, CDKN1A). Epigenetic changes induced by these metals may persist after pregnancy, increasing the risk of metabolic, neurodegenerative, and neoplastic disorders in offspring later in life.

Several toxic metal(loid)s also function as endocrine disruptors by binding to nuclear receptors, such as estrogen receptors (ER), glucocorticoid receptors (GR), and thyroid hormone receptors (THR), thereby impairing the hormonal regulation necessary for maintaining pregnancy. Additionally, an imbalance in the concentrations of oncoelements may promote low-grade inflammation by activating nuclear factor kappa-light-chain-enhancer of activated B cells (NF-κB) and mitogen-activated protein kinase (MAPK) signaling pathways. This inflammatory state further amplifies oxidative stress and compromises placental function.

In contrast, selenium and zinc contribute to maintaining proper methylation balance by regulating the one-carbon cycle and enhancing DNA methyltransferase activity. These actions may support genomic stability and reduce susceptibility to aberrant silencing of genes involved in apoptosis, cell-cycle regulation, and immune function.

### 3.1. Selected Toxic Elements—Arsenic, Cadmium, Lead

Arsenic (As) is a toxic metalloid commonly found in the environment in both organic and inorganic forms. It occurs naturally in rocks, soil, water, air; it is also present in plants and animals. The primary source of human exposure is through the consumption of contaminated drinking water, particularly in regions with naturally high arsenic concentrations in groundwater, such as Bangladesh, India, Mexico, China, and Chile [10]. After absorption, arsenic is metabolized in the liver via methylation and excreted mainly as dimethylarsinic acid (DMA) and monomethylarsonic acid (MMA). However, incomplete methylation may result in the formation of more toxic intermediates, such as MMA(III), which exhibits high reactivity and genotoxic potential. Although arsenic is not classified as a classical mutagen, its carcinogenic properties are mediated through multiple mechanisms, including oxidative stress, epigenetic deregulation (alterations in DNA methylation and histone modifications), mitochondrial dysfunction, and inhibition of DNA repair. Arsenic binds with high affinity to thiol and selenol groups, leading to the inactivation of antioxidant enzymes, such as thioredoxin reductase. Disruption of selenium-dependent enzymes contributes to increased oxidative stress and subsequent cellular damage [19].

The International Agency for Research on Cancer (IARC) has classified inorganic arsenic as a Group 1 carcinogen, particularly in relation to bladder, skin, and lung cancers [20]. Chronic, low-level arsenic exposure often does not follow a typical dose–response relationship, complicating risk assessment. This underscores the importance of coexisting factors such as nutritional deficiencies (e.g., selenium, folate) and genetic polymorphisms (e.g., in the *AS3MT* gene), which affect arsenic methylation efficiency. Combined exposure to arsenic and other heavy metals (e.g., cadmium, lead) or endocrine-disrupting chemicals (EDCs) may exacerbate oxidative stress and epigenetic alterations [21,22].

Exposure typically occurs via ingestion of food or water contaminated with arsenic-containing pesticides, herbicides, or insecticides. Inhalation is another significant route, especially in occupational settings. Global dietary intake of total arsenic typically ranges from 20 to 300 µg/day [23,24]. Mechanisms of arsenic toxicity include oxidative stress, DNA damage, impaired DNA repair, epigenetic alterations, and disruption of hormonal signaling and metabolic processes [25].

Cadmium (Cd) is a heavy metal with a very long biological half-life and significant bioaccumulation potential. The main routes of exposure include ingestion of contaminated food and water and inhalation, especially via cigarette smoke. Anthropogenic activities such as mining, fertilizer application, and sewage irrigation contribute significantly to environmental cadmium contamination, allowing its entry into the food chain. Once absorbed, cadmium binds to albumin and metallothionein and accumulates primarily in the liver and kidneys, but also in muscles and bones. Cadmium is excreted via urine and feces, and its biological half-life in blood can extend over several years. Chronic exposure may result in damage to the kidneys, bones, intestines, liver, and reproductive system, and may also lead to anemia [26].

Cadmium promotes the production of reactive oxygen species (ROS) and reactive nitrogen species (RNS), depletes intracellular glutathione (GSH), and inhibits the activity of antioxidant enzymes. It competes with essential metal ions (Zn^2+^, Ca^2+^, Fe^2+^, Cu^2+^) for binding sites on metalloproteins, disrupting physiological processes such as bone mineralization, iron transport, and nervous system function, leading to nephrotoxicity [27,28].

Although cadmium is not directly mutagenic, it induces genotoxicity indirectly by generating ROS and inhibiting DNA repair systems, including nucleotide excision repair (NER) and base excision repair (BER). These effects alter the expression of genes involved in cell proliferation and differentiation [29]. The IARC classifies cadmium as a Group 1 human carcinogen, primarily based on evidence of increased lung cancer risk in occupational exposure. Acute inhalation toxicity thresholds include 0.5 mg/m^3^ for cadmium oxide fumes, and 0.25 mg/m^3^ for single exposures lasting one hour [20].

Lead (Pb) is a toxic heavy metal that has no physiological function in the human body. It is not biodegradable and has the ability to accumulate in the tissues of living organisms. After being absorbed through the respiratory or digestive tract, it primarily binds to erythrocytes and is then distributed to soft tissues (brain, kidneys, liver) and bones, which are its main storage sites. Small amounts of lead can also penetrate the skin, especially in the case of occupational exposure. The biological half-life of lead in rapidly replaced tissues, such as blood and soft tissues, is approximately 30 days, while in slowly replaced tissues, such as bones, teeth, and nails, it ranges from 2.3 to 27 years. Lead compounds are eliminated from the body in urine, feces, sweat, and breast milk [30].

At the cellular level, lead mimics calcium ions (Ca^2+^), entering neurons and muscle cells via calcium channels and Ca^2+^-dependent transporters. This disrupts nerve conduction, neurotransmitter release, and smooth muscle contraction [31]. Lead induces oxidative stress by stimulating ROS production and inhibiting the activity of antioxidant enzymes (e.g., superoxide dismutase, catalase, glutathione peroxidase), which leads to lipid peroxidation and damage to DNA and proteins [13,32]. Additionally, it disrupts heme biosynthesis, resulting in the accumulation of neurotoxic intermediates and the development of anemia [33,34].

Chronic lead exposure is associated with multisystem toxicity, including neurocognitive impairment, renal dysfunction, hematologic effects (via disruption of heme biosynthesis), and cardiovascular risk. In pregnancy, lead is of particular concern due to placental transfer and its established association with adverse neurodevelopmental outcomes in offspring [35].

Lead is classified by IARC as probably carcinogenic to humans (Group 2A) [20]. Current consensus holds that no blood lead level is considered safe, particularly during pregnancy. Concentrations as low as 2–5 µg/dL can impair fetal neurodevelopment [30].

### 3.2. Selected Elements with Mainly Protective Effects—Selenium, Zinc, Copper

Zinc (Zn) is an essential trace element that plays a critical role in numerous physiological processes, including the function of the cardiovascular, skeletal, reproductive, and immune systems. It serves as a cofactor for enzymes involved in protein and carbohydrate metabolism and supports immune function by regulating T-cell proliferation, cytokine production, and natural killer (NK) cell activation [36].

From a molecular perspective, zinc contributes to genome stability. It acts as a cofactor for DNA and RNA polymerases, ligases, and endonucleases involved in DNA replication and repair. Additionally, it serves as an antioxidant by stabilizing cell membranes and proteins containing thiol groups, thereby reducing their susceptibility to oxidative damage. Zinc deficiency impairs the regulation of inflammatory and immune responses and may contribute to the development of skin conditions, allergic reactions, and alopecia. Conversely, excessive zinc intake can interfere with the absorption of other trace elements, particularly copper and iron, leading to hematopoietic dysregulation and neurological dysfunction [36,37,38].

Chronic exposure to high levels of zinc may cause anemia, granulocytopenia, bone marrow suppression, and neurological symptoms such as spasticity, sensory ataxia, and imbalance. Acute zinc toxicity can result from oral ingestion of 1–2 g of elemental zinc, leading to gastrointestinal bleeding, renal and hepatic impairment, and severe thrombocytopenia, which in extreme cases may be fatal. A condition known as metal fume fever (also referred to as zinc fever or foundry fever) is a non-specific systemic response triggered by the inhalation of metal vapors with particle sizes < 1 μm, including zinc, copper, manganese, nickel, chromium, and cadmium [39].

Although zinc is not classified as a carcinogen, disturbances in its homeostasis can modulate cellular susceptibility to carcinogenic factors [29].

Copper (Cu) is an essential trace element that functions as a cofactor in numerous redox reactions. In the human body, it plays a crucial role in the mitochondrial respiratory chain as a component of cytochrome c oxidase, facilitating efficient ATP production. In combination with zinc, it forms Cu/Zn superoxide dismutase (Cu/Zn-SOD), an enzyme critical for the detoxification of superoxide anions. Additionally, copper participates in connective tissue synthesis and angiogenesis (e.g., via lysyl oxidase), as well as in neurotransmitter metabolism [40].

Maintaining copper homeostasis requires tightly regulated intracellular and extracellular transport. Both copper deficiency and excess can lead to dysfunction in multiple organ systems. Dietary copper deficiency may result in anemia, leukopenia, neutropenia, and pancytopenia, as well as osteoporosis, increased fracture risk, and neurodegenerative conditions, including Menkes disease and hereditary peripheral neuropathies. Copper imbalance has also been implicated in the pathogenesis of neurodegenerative diseases such as Parkinson’s, Alzheimer’s, and Huntington’s diseases [41,42].

Acute copper toxicity is rare and typically associated with accidental or intentional ingestion of high doses. It may lead to serious clinical outcomes such as hemolytic anemia, gastrointestinal bleeding, hepatotoxicity, nephrotoxicity, and, in severe cases, death. Gastrointestinal symptoms may occur even with doses below 1 g [43,44].

Copper compounds are classified by the International Agency for Research on Cancer (IARC) as Group 3—not classifiable as to carcinogenicity to humans. However, chronic metabolic disorders such as Wilson’s disease support copper’s involvement in the pathophysiology of chronic diseases where oxidative stress is a major contributing factor [20].

Selenium (Se) is an essential trace element and is present in the body in the form of over 25 known selenoproteins. The most important of these are glutathione peroxidases (GPx), which catalyze the reduction of lipid peroxides and H_2_O_2_, protecting cell membranes and DNA from oxidative damage. Other important enzymes include thioredoxin reductase (TrxR), which is crucial for maintaining the reduced form of proteins and regulating thiol-dependent signaling pathways, as well as iodothyronine deiodinases, which are responsible for the conversion of thyroid hormones (T_4_ → T_3_), which is important for metabolism and nervous system development [45,46].

At the molecular level, selenium enhances antioxidant defense systems, acting synergistically with vitamin E and other antioxidants. It modulates the balance between pro-inflammatory and protective responses and regulates apoptosis pathways through the modulation of Bcl-2 family proteins and caspase activity. Additionally, selenium exhibits chemopreventive potential against certain types of cancer [47].

Selenium deficiency can increase susceptibility to oxidative stress, impair immune responses, and contribute to cardiovascular and thyroid dysfunction [48,49]. Conversely, chronic intake exceeding recommended dietary levels (e.g., 55 µg/day for adults and 60 µg/day for pregnant women in the Polish population) may lead to selenium toxicity (selenosis). Adverse effects may include gastrointestinal disturbances, hepatotoxicity, neurotoxicity, and dermatological or mucosal lesions [50].

Selenium supplementation has demonstrated protective effects against cisplatin-induced nephrotoxicity in cancer patients. Similar benefits have also been observed in individuals undergoing chemoradiotherapy [51].

Selenium compounds are classified by the International Agency for Research on Cancer (IARC) as Group 3—not classifiable as to carcinogenicity to humans. Nevertheless, both selenium deficiency and excess may influence the risk of oxidative stress-related diseases [20].

Toxic metals often mimic or displace essential elements from their enzymatic binding sites, thereby disrupting the activity of key metalloenzymes. Cadmium competes with zinc for binding sites within metallothioneins and zinc-dependent enzymes, resulting in functional zinc depletion, redox imbalance, and reduced activity of superoxide dismutase (SOD). Lead interferes with calcium-mediated signaling by mimicking Ca^2+^ ions, thereby disrupting neurotransmission, muscle contractility, and calcium-regulated gene expression. Arsenic, in its arsenate form, can substitute for phosphate in oxidative phosphorylation reactions, leading to mitochondrial dysfunction and impaired ATP production.

Collectively, these interactions converge on interconnected redox-inflammatory, mitochondrial, and epigenetic nodes relevant to placental function and fetal growth [2,5,7,8].

## 4. Protective Functions of the Placenta in the Context of Maternal Oncoelement Exposure

The placenta is a multifunctional organ essential for fetal development and maternal adaptation to pregnancy. Beyond nutrient and gas exchange, it acts as a selective barrier against environmental toxicants [52]. Efficient oxygen and nutrient delivery to the fetus relies on proper vasculogenesis and angiogenesis within the placenta. The processes of neovascularization and the establishment of functional placental circulation are regulated by a fine-tuned balance between proangiogenic and antiangiogenic signals. Key factors in this process include placental growth factor (PlGF), vascular endothelial growth factor (VEGF), and its soluble type 1 receptor, soluble fms-like tyrosine kinase-1 (sFlt-1). These molecules, synthesized by trophoblast cells, orchestrate the growth of both the placenta and fetus, directly influencing the quality of vascularization and the efficiency of nutrient transport within the maternal–fetal interface [53]. Moreover, recent studies highlight significant interindividual variability in placental vascular development, influenced by genetic polymorphisms in angiogenic and oxidative stress-related genes [8].

In addition to its metabolic and transport functions, the placenta also acts as an endocrine gland and an immunological organ. It synthesizes a wide range of steroid and peptide hormones, including human chorionic gonadotropin (hCG), human placental lactogen (hPL), progesterone, and estrogens, which contribute to maternal physiological adaptation to pregnancy.

Trophoblast cells express human leukocyte antigen G (HLA-G), which contributes to immune tolerance at the maternal–fetal interface and may modulate local antiviral immune responses. Abnormal expression of HLA antigens may contribute to recurrent pregnancy loss, implantation failure in in vitro fertilization procedures, and preeclampsia. The syncytiotrophoblast layer also functions as an antimicrobial barrier, producing antiviral and antibacterial peptides that limit the transplacental transmission of pathogens such as *Cytomegalovirus*, *Listeria monocytogenes*, and *Toxoplasma gondii* [52].

The placenta is a dynamic barrier between the maternal environment and the fetus; however, its protective capacity is not absolute. Essential elements for fetal development, including selenium (Se), zinc (Zn), and copper (Cu), are selectively transported via specific metal transporters such as zinc transporter 1 (ZnT1), Zrt- and Irt-like protein 8 (ZIP8), and ATPase copper-transporting A/B (ATP7A/B). Conversely, toxic metals: cadmium (Cd), lead (Pb), arsenic (As), and mercury (Hg) can cross the placental barrier by mimicking physiological ions or by damaging trophoblast cell membranes through oxidative stress. Other harmful substances, such as ethanol, polycyclic aromatic hydrocarbons, pesticides, and compounds found in tobacco smoke, can also be transferred to the fetus. In addition to chemical structure and lipophilicity, the efficiency of placental transfer of trace elements is modulated by individual genetic makeup and placental epigenetic mechanisms. DNA methylation and histone modifications may regulate the expression of metal transporters and oxidative stress pathways, thereby altering susceptibility to toxic exposure and influencing fetal programming [54].

Exposure to toxic metal(loid)s induces excessive production of reactive oxygen species (ROS) in placental mitochondria and microsomes. While moderate levels of ROS are essential for physiological signaling and trophoblast invasion, excessive ROS production leads to lipid peroxidation, DNA and protein damage, and the activation of proinflammatory pathways. The placental antioxidant system including superoxide dismutase (SOD), catalase (CAT), glutathione peroxidase (GPx), and thioredoxin reductase (TrxR) is impaired by chronic exposure to cadmium and lead. Oxidative imbalance activates transcription factors such as NF-κB and AP-1, enhancing the expression of proinflammatory cytokines and promoting endothelial dysfunction. This cascade results in placental hypoxia, exacerbates oxidative stress, and triggers a vicious cycle of cellular injury [52].

From a molecular biology perspective, the placenta functions both as a target and a mediator of the toxic effects of heavy metals. Disruption of oxidative–antioxidative homeostasis and epigenetic regulation serves as a mechanistic link between maternal exposure, placental dysfunction, and adverse pregnancy outcomes. Quantification of metal concentrations in placental tissue, along with biomarkers of oxidative stress, provides valuable insight into the mechanisms of fetal exposure and developmental programming, consistent with the concept of the Developmental Origins of Health and Disease (DOHaD) [8].

## 5. Maternal Blood Concentrations and Pregnancy Outcomes

### 5.1. Selenium

During pregnancy, maternal blood selenium concentrations decrease significantly, particularly in the second and third trimesters. This phenomenon is associated with active placental transport of selenium to the fetus, thereby helping to ensure adequate antioxidant protection during development [45].

Selenium deficiency has been implicated in the etiology of neural tube defects. In women with pregnancy-induced hypertension, significantly lower selenium concentrations have been observed as early as the first trimester, compared to normotensive pregnant women [55]. Low maternal selenium levels also contribute to increased oxidative stress, which may result in adverse outcomes such as miscarriage, premature rupture of membranes, preterm birth, and intrauterine growth restriction (IUGR) [56,57]. In cases of preterm birth and IUGR, elevated selenium levels have been detected in placental tissue when compared with placentas from uncomplicated pregnancies. This finding may reflect a compensatory response or impaired selenium transport to the fetus [58]. There may also be an association between abnormal maternal selenium status and conditions such as preeclampsia, thyroid dysfunction during and after pregnancy, impaired glucose tolerance, gestational diabetes, and intrahepatic cholestasis [57,59].

The literature includes a case report of severe chronic selenium toxicity in a pregnant woman, presenting with nausea, vomiting, limb paresthesia, fatigue, and alopecia accompanied by nail loss. Following hospitalization, the pregnancy concluded at term without complications, and no congenital anomalies were observed in the newborn [59].

### 5.2. Zinc

The concentration of zinc in the plasma of pregnant women gradually decreases from the first to the last trimester, which is explained by the active transport of the element from the mother’s body to the developing fetus [60]. The simultaneous accumulation of cadmium in the umbilical cord blood and placenta may further interfere with the effective transfer of zinc to fetal tissues [28].

The literature does not provide clear data confirming the embryotoxic or teratogenic effects of zinc in people occupationally exposed to this element. Nevertheless, it is indicated that the potential risk to pregnant women does not result directly from the presence of zinc ions, but from the occurrence of metallic fever as a reaction to inhalation of metal fumes [39]. Some studies suggest that high zinc concentrations in the mother’s blood (e.g., ≥896.59 μg/L) may mitigate the negative effects of prenatal mercury exposure. Since exposure to mercury during pregnancy may increase the risk of preterm birth, an adequate supply of zinc in the diet may have a protective effect [61].

Zinc deficiency in pregnant women may exacerbate placental inflammation, which is considered one of the key risk factors for preterm birth [62]. Reduced zinc concentrations have also been observed in women who have experienced miscarriage, premature rupture of membranes, or IUGR [63]. Zinc supplementation in women with zinc deficiency may have a protective effect, reducing the risk of preeclampsia and pulmonary embolism [64].

### 5.3. Copper

Copper plays an important biological role in fetal growth and development. In pregnant women, its concentration in plasma is significantly elevated compared to preconception levels and returns to baseline after delivery. Copper deficiency in the pregnant woman’s diet and abnormal maternal body weight can lead to structural and metabolic abnormalities in the embryo and fetus [60]. Lower copper concentrations are observed in women with infertility, after miscarriages, and in cases of intrauterine growth restriction (IUGR) [65].

Elevated serum copper levels in pregnant women are associated with higher fasting glucose concentrations and higher glucose concentrations after oral glucose loading, which may increase the risk of gestational diabetes [66]. Wilson’s disease, a metabolic disorder leading to copper accumulation in tissues, is often associated with fertility disorders and recurrent miscarriages. However, there are reports of successful births in patients treated with zinc sulfate, which reduces copper absorption in the intestines and its deposition in organs [67].

Women without Wilson’s disease but with high copper and low zinc levels (e.g., as a result of environmental exposure) also have a higher risk of pregnancy pathologies such as miscarriages, preeclampsia, HELLP syndrome, and IUGR [62,65]. Elevated copper levels in peripheral blood may also be an independent risk factor for preterm birth. In the first trimester, copper exposure may be associated with lipid disorders (hypercholesterolemia, hypertriglyceridemia), which indirectly increase the risk of preterm birth [14]. Measuring serum copper levels in the first trimester (10–14 weeks) may serve as a biomarker for the risk of pregnancy-induced hypertension, as copper deficiency is associated with reduced antioxidant enzyme activity [62].

### 5.4. Arsenic

Exposure of pregnant women to arsenic poses a significant risk to both the health of the mother and the developing fetus. The effects can vary, ranging from metabolic disorders in the mother, through adverse effects on fetal development, to the occurrence of birth defects. Given the permeability of arsenic through the placental barrier and its complex mechanisms of toxicity, preventive measures are recommended. Peripheral blood reflects current exposure (due to its short half-life), while umbilical cord blood better reflects fetal exposure. The presence of arsenic in maternal blood is associated with an increased risk of adverse pregnancy outcomes, which may be explained by mechanisms such as oxidative stress, epigenetic dysregulation, and endocrine disorders. The result may be impaired placental function and reduced fetal growth [17,68].

Epidemiological studies suggest that higher concentrations of arsenic in the mother’s blood are associated with lower birth weight, an increased incidence of intrauterine growth restriction (IUGR) and small for gestational age (SGA) infants, as well as an increased risk of preterm birth. These relationships tend to be stronger when the more toxic methylated MMA% fraction accounts for a larger proportion of the total arsenic pool, which is associated with weaker methylation efficiency [15].

High concentrations of this element are also suggested to contribute to spontaneous miscarriages, placental insufficiency, gestational hypertension/preeclampsia, and congenital defects in the fetus [15]. Sensitivity to arsenic may depend on the window of exposure (often stronger effects in the first and second trimesters) and nutritional factors. Adequate folate and selenium status may support methylation and detoxification of arsenic, which may mitigate its toxic effects. In contrast, micronutrient deficiencies and co-exposure to tobacco smoke may exacerbate the harmful effects of arsenic [68].

### 5.5. Lead

Pregnant women may be exposed to lead through environmental sources (e.g., air, soil, and water pollution), occupational exposure, and smoking. The lead content in a single cigarette can range from 0.4 to 1.3 µg, and passive smoking is also associated with a significant risk of increased lead concentrations in maternal and umbilical cord blood [31]. Women exposed to lead, especially smokers or those living in polluted areas, often also have elevated cadmium concentrations [11]. Calcium deficiency during pregnancy may exacerbate the toxic effects of lead on the fetus, as lead competes with calcium for binding sites in bones and enzymes [31]. Lead easily crosses the placenta, and its neurotoxic effects are particularly harmful to the developing fetal nervous system. Furthermore, fetal hemoglobin has a higher affinity for lead than adult hemoglobin [66]. Lead stored in the mother’s bones can be released into the circulation during pregnancy and constitutes a secondary source of fetal exposure [11,17].

Lead’s ability to cross both the placental and blood–brain barriers makes it a significant risk factor for neurological disorders, including delayed cognitive and motor development [33,34]. Although some research suggests that low maternal blood lead levels (<10 µg/dL) may not significantly increase the risk of preterm birth, other studies indicate that higher exposure—measured in urine samples—corresponding to the highest tertile of exposure (≥4.70 µg/g creatinine), is associated with approximately a 73% increased risk of preterm PROM, i.e., premature rupture of membranes [69].

Umbilical cord blood lead concentrations exceeding 0.15 µmol/L may be associated with neurodegenerative changes in the fetal central nervous system [68]. Calcium supplementation during pregnancy may reduce the transfer of lead into fetal circulation. In addition, zinc and magnesium exert a protective effect on the N-methyl-D-aspartate (NMDA) receptor in the brain, which plays a key role in learning and memory processes, and their presence may reduce lead-induced neurotoxicity [34].

### 5.6. Cadmium

Women’s bodies are more susceptible to cadmium than men’s due to hormonal and metabolic differences, as well as a greater need for micronutrients during pregnancy. Environmental or occupational exposure to cadmium can negatively affect female reproductive health by disrupting hormonal pathways and generating oxidative stress [29].

Women with recurrent miscarriages had higher cadmium concentrations in their peripheral blood compared to women with no history of pregnancy loss. Elevated cadmium levels were also detected in the blood and placentas of women who had miscarried, compared to women in their first trimester of pregnancy and after delivery [70]. However, no significant association was found between low cadmium concentrations (approx. 0.32–0.34 μg/L) and the occurrence of ectopic pregnancy [71].

The toxic effects of cadmium include placental damage, which may lead to fetal development disorders and potential birth defects such as brain hernia or hydrocephalus. The teratogenicity of cadmium is partly related to its ability to displace zinc from binding sites in enzymes, resulting in functional zinc deficiency and inhibition of key enzymes responsible for DNA synthesis and stability, including those involved in thymine incorporation [20]. Increased cadmium concentrations in the placenta disrupt calcium and vitamin D metabolism, which may affect fetal tissue growth and mineralization [32]. Cadmium present in maternal tissues, the placenta, and fetal fluids may also increase the risk of developing preeclampsia [72,73].

Oxidative stress caused by cadmium is considered one of its main toxic mechanisms. The antioxidant effects of zinc and selenium may offer protection against cadmium-induced damage. Additionally, exposure to cadmium during pregnancy, especially in women of lower socioeconomic status has been associated with an increased risk of pregnancy-induced hypertension [73]. Studies have confirmed a significant association between cadmium levels in maternal blood and the occurrence of intrauterine growth restriction (IUGR) and preterm birth. Cadmium detected in the plasma of women with preeclampsia correlates with fetal growth restriction [72]. The accumulation of cadmium in the mother’s body, in blood, placenta, and fetal membranes increases with the number of cigarettes smoked [74].

Higher cadmium concentrations in maternal blood during the first and second trimesters are considered a marker of increased risk for premature birth (<34 weeks of gestation). Multiple studies confirm a positive correlation between cadmium presence in maternal blood and the risk of preterm delivery [18].

Table 1 presents the main pathways of action of oncoelements, molecular mechanisms, and clinical significance for pregnancy. This summary is a compilation of chapters on molecular mechanisms (Chapter 3) and the impact of element concentrations in maternal blood on pregnancy outcomes (Chapter 5).

Overall, the evidence summarized in this section is predominantly observational, and reported associations vary in magnitude across studies. Heterogeneity is likely driven by differences in exposure matrices (maternal blood vs. urine vs. cord blood), timing of sampling (trimester-specific variability), adjustment for key confounders (notably smoking, diet, and socioeconomic status), and regional exposure profiles. For several outcomes (e.g., IUGR/SGA for arsenic and cadmium; neurodevelopmental endpoints for lead), the direction of association is relatively consistent, whereas other links (e.g., copper and hypertensive disorders; selenium and PROM) remain mixed and should be interpreted cautiously.

## 6. Cord Blood as a Biomarker of Prenatal Exposure to Oncoelements

Umbilical cord blood is a unique biological matrix that reflects both the physiological status of the fetus and its prenatal environmental exposures. Its biochemical composition provides valuable insight into nutrient transport, oxidative-inflammatory processes, and fetal exposure to potentially harmful substances, including toxic metals and endocrine-disrupting chemicals (EDCs). Cord blood is also a rich source of hematopoietic stem cells (HSCs) and can be collected in a non-invasive, safe, and ethically acceptable manner, making it particularly suitable for both clinical and research applications. Studies indicate that factors such as maternal age and neonatal birth weight do not significantly affect cord blood quality, supporting its utility across diverse populations [16].

From the perspective of environmental toxicology, umbilical cord blood serves as a direct and sensitive biomarker of fetal exposure to trace and toxic elements. These include both protective (e.g., selenium, zinc, copper) and toxic oncoelements (e.g., cadmium, lead, arsenic), which are transferred through the placenta to varying extents. The concentrations of these elements in cord blood reflect not only maternal exposure but also placental transport capacity and accumulation efficiency.

Cord blood analysis, particularly when combined with oxidative stress biomarkers, offers insight into the mechanisms by which early-life exposure to metals may influence neonatal anthropometric parameters and developmental outcomes [75,76].

Toxic metals in cord blood have been shown to promote oxidative and nitrosative stress, resulting in biomolecular damage and epigenetic dysregulation. They can also suppress antioxidant enzyme activity and modulate redox-sensitive gene expression through transcription factors such as NF-κB and Nrf2. Moreover, these elements may disrupt epigenetic regulation, affecting DNA methylation and histone modification, which can result in lasting changes to genes involved in fetal growth, metabolism, and immune function.

Numerous studies report inverse associations between toxic metal(loid) concentrations in cord blood and neonatal indicators such as birth weight, body length, and head circumference. These findings support the role of prenatal metal exposure in fetal growth restriction and in developmental programming associated with long-term risk of chronic diseases [76].

EDCs such as phthalates, phenols, and polyfluoroalkyl substances also accumulate in fetal tissues and interfere with endocrine signaling. In mothers, they have been associated with pregnancy complications including miscarriage, preterm birth, and preeclampsia. In fetuses, EDC exposure may contribute to intrauterine growth restriction, low birth weight, and altered hormone homeostasis. While maternal EDCs are typically measured in urine, fetal exposure is assessed using umbilical cord blood [77,78].

Despite its utility in environmental and perinatal research, the interpretation of cord blood data is subject to several methodological limitations:

- Analytical variability—measurement accuracy may vary depending on laboratory techniques (e.g., inductively coupled plasma mass spectrometry (ICP-MS) vs. atomic absorption spectrometry (AAS)), sample preparation, and instrumentation [5,16,76].
- Sampling and storage protocols—inconsistencies in the timing of collection, handling procedures, and biobanking practices can influence element concentrations [16,79].
- Lack of standardized reference values—clinical or toxicological thresholds for many trace elements in cord blood remain poorly defined, limiting diagnostic interpretation [76].
- Mixture effects and confounding factors—simultaneous exposure to multiple metals and pollutants complicates attribution of specific effects to individual agents [2,3,11].
- Limited longitudinal data—few studies have correlated cord blood concentrations with long-term child health outcomes, which weakens causal inference [11,76].

In response to these challenges, especially the issue of mixture effects, recent studies have employed advanced statistical approaches for multi-pollutant exposure analysis. Methods such as Weighted Quantile Sum (WQS) regression, Bayesian Kernel Machine Regression (BKMR), and principal component analysis (PCA) enable the assessment of combined effects while accounting for collinearity and interactions [80].

These techniques can help identify exposure mixtures associated with outcomes like fetal growth restriction or oxidative stress. However, their application in perinatal research remains limited, and further methodological standardization is needed to improve reproducibility and comparability across studies [3,5,81,82].

Nevertheless, cord blood remains a critical biomatrix for assessing prenatal exposure to both protective and toxic elements. Compared to other matrices such as maternal blood, urine, or placental tissue, cord blood provides a direct snapshot of fetal exposure, representing the net result of placental transfer. However, it does not reflect maternal body burden across time. In contrast, placental tissue offers insight into elemental accumulation and transport mechanisms, while maternal fluids are more suitable for longitudinal monitoring throughout pregnancy. Therefore, combining multiple biological matrices may enhance the characterization of the prenatal exposome, although further research is needed to validate their comparative effectiveness in diverse populations [16,76,79].

Table 2 provides a comparative overview of the maternal and fetal effects of individual oncoelements discussed in Section 3, Section 4, Section 5 and Section 6. It integrates clinical outcomes, biological activity (protective vs. toxic), and key modifying factors such as micronutrient status and environmental exposures.

The comparative layout of Table 2 supports the identification of key oncoelements that may warrant targeted screening or nutritional interventions, particularly among high-risk maternal populations.

Table 3 summarizes reported associations between trace element status and maternal or fetal outcomes, highlighting the direction and consistency of effects, distinguishing between protective and toxic elements, and mapping exposure pathways via relevant biomarkers and mechanistic bridges.

The complexity and heterogeneity of evidence across studies is reflected in the “mixed” findings and limited consistency for certain outcomes. Mixed findings most commonly reflect differences in exposure assessment, timing, and residual confounding rather than clear biological contradiction. However, the trends observed for elements such as selenium, arsenic, and cadmium demonstrate stronger and more reproducible links, particularly in relation to oxidative stress, endocrine disruption, and redox imbalance.

## 7. Future Directions and Clinical Implications

Despite growing evidence linking trace element status in pregnant women to pregnancy outcomes and newborn anthropometry, the current body of research remains predominantly observational and limited by methodological and conceptual heterogeneity. Future studies should aim to more precisely define dose–response relationships and establish threshold concentrations that distinguish between deficiency and toxicity, particularly for selenium, zinc, copper, cadmium, and lead. Although cross-sectional studies dominate the field, findings are often inconsistent due to differences in population characteristics, sample matrices, and timing of biomarker assessment. In addition, the majority of studies are limited by small sample sizes, lack of harmonized methodologies, and insufficient adjustment for confounders such as maternal diet or genetic variability.

Further investigations should incorporate molecular biomarkers of oxidative stress, mitochondrial dysfunction, and epigenetic regulation to better elucidate the mechanistic pathways linking trace element imbalances to placental dysfunction and fetal development. Prospective, longitudinal cohort studies with repeated biomarker measurements across different trimesters are particularly needed to identify critical windows of susceptibility and to strengthen causal inference. Combining these approaches may facilitate the identification of early molecular signatures predictive of adverse pregnancy outcomes.

From a clinical standpoint, routine monitoring of micronutrient concentrations, especially selenium and zinc may be considered in high-risk groups, such as women with pregnancy-induced hypertension, gestational diabetes, or intrauterine growth restriction. However, recommendations for widespread screening or supplementation should remain cautious until supported by stronger interventional evidence. Nutritional strategies aimed at supporting antioxidant balance should be tailored to local exposure profiles to avoid both deficiency and excess.

In regions affected by environmental contamination (e.g., in soil, air, or drinking water), screening for toxic elements such as cadmium, lead, or arsenic may be warranted, particularly among women with known risk factors such as tobacco use or low socioeconomic status [1,2,7,78].

From a public health perspective, priority should be given to reducing environmental emissions of toxic metals, ensuring the safety of food and drinking water, and increasing awareness of dietary sources of essential micronutrients. Environmental exposure assessments, though still evolving, may complement prenatal care and risk stratification in the future. However, their integration into routine practice will require further validation, cost–benefit analysis, and public health consensus. Raising public awareness about environmental health risks and implementing targeted health education initiatives remain essential strategies for minimizing preventable exposures to environmental pollutants [12,82,83,84].

## 8. Conclusions

The balance of trace elements in maternal circulation plays a critical role in supporting antioxidant defenses, hormone synthesis, and epigenetic regulation during pregnancy. Both deficiency and excess of micronutrients, such as selenium, zinc, and copper may contribute to pregnancy complications and suboptimal neonatal outcomes. Likewise, prenatal exposure to toxic elements including cadmium, lead, and arsenic has been associated with oxidative damage, impaired placental function, and adverse fetal development.

A growing body of observational evidence suggests that the effects of oncoelements result from complex and often non-linear interactions, shaped by both environmental burden and maternal nutritional status. However, more robust data, particularly from prospective, mechanistic, and interventional studies are needed to confirm causality and inform clinical guidelines.

From a practical perspective, selective monitoring of trace elements in high-risk populations may prove beneficial, especially when integrated with individualized nutritional strategies. Preventive actions aimed at reducing environmental exposures and securing the safety of food and water sources remain essential public health priorities.

Understanding the biological and clinical impact of prenatal oncoelement exposure may support strategies for reducing the burden of chronic diseases across generations, consistent with the Developmental Origins of Health and Disease (DOHaD) framework. In the long term, interdisciplinary collaboration between environmental science, molecular biology, and perinatal medicine will be key to translating this knowledge into effective clinical and public health interventions.

## Figures and Tables

**Table 1:** Oncoelements—molecular mechanisms and significance for pregnancy in the context of maternal exposure.

Oncoelements		Main Pathways	Molecular Mechanisms	Significance for Pregnancy
Elements with protective properties	Se	Antioxidant defense, thyroid hormone metabolism, apoptosis	GPx and TrxR activation, T_4_→T_3_ thyroid hormone conversion, Bcl-2 modulation, synergism with vitamin E, cytokine regulation, genome stability maintenance, and NK cell-mediated immunity	Protects against oxidative damage, supports fetal thyroid and immune function, lower maternal Se status has been reported in association with preeclampsia, thyroid dysfunction, gestational diabetes, and intrahepatic cholestasis; associated with newborn birth weight [54,55,56,57,58,59].
	Zn	DNA repair, immunity, redox balance and DNA/RNA integrity	Polymerase function, stabilization of SOD, regulation of pro- and anti-inflammatory cytokines, promotion of DNA integrity and immune defense	Supports immune system development and fetal growth, may support antioxidant defenses within physiological range, Zn deficiency is associated with LBW, prematurity, and preeclampsia [60,61,62,63,64].
	Cu	Mitochondrial respiration, redox reactions, angiogenesis	Cofactor for cytochrome c oxidase, Cu/Zn-SOD, lysyl oxidase; involved in neurotransmission and signaling pathways during fetal neurodevelopment	Essential for ATP production and vascular development; interacts with zinc-dependent antioxidant systems. Altered Cu levels are linked to infertility, miscarriages, preeclampsia, intrauterine growth restriction (IUGR), HELLP syndrome, preterm birth, and gestational diabetes [14,60,65,66,67].
Elements with toxic effects	As	Oxidative stress, epigenetic deregulation, mitochondrial dysfunction	TrxR inhibition, alterations in DNA methylation, ROS generation, impairment of ATP production and oxidative phosphorylation	Associated with IUGR, LBW, SGA, preeclampsia, placental insufficiency, metabolic disorders in offspring, and lower Apgar scores [15,17,68].
	Pb	Calcium signaling, oxidative stress, neurotoxicity	Calcium mimicry, inhibition of antioxidant enzymes, induction of DNA damage and oxidative stress	Causes fetal neurotoxicity and anemia; impairs placental perfusion. Associated with preterm birth, PROM, and reduced birth weight [11,31,69,74].
	Cd	ROS/RNS generation, DNA repair inhibition, calcium signaling	Zinc competition, inhibition of NER/BER pathways, glutathione (GSH) depletion, disruption of mitochondrial function, and activation of MAPK and NF-κB signaling pathways	Increases risk of miscarriage, preeclampsia, and developmental defects. Associated with IUGR, LBW, and preterm birth [18,23,29,70,71,72,73,74].

Se—Selenium; Zn—Zinc; Cu—Copper; As—Arsenic; Pb—Lead; Cd—Cadmium.

**Table 2:** Comparison of the effects of selected oncoelements on maternal and fetal parameters.

Oncoelements	Maternal Parameters	Fetal Parameters	Type of Effect	Source of Exposure/Modifying Factor
Se	Low status: pregnancy-induced hypertension, intrahepatic cholestasis of pregnancy, gestational diabetes mellitus (GDM)	Low status: low birth weight (LBW), intrauterine growth restriction (IUGR), small for gestational age (SGA), preterm birth (PTB)	Protective (adequate status)/adverse in deficiency	Deficiency, interactions with cadmium
Zn	Low status: preeclampsia, infections, anemia	Low status: LBW, PTB, IUGR	Protective (adequate status)/adverse in deficiency	Deficiency, antagonism by cadmium
Cu	High status/excess: GDM, preeclampsia, hypertension	High status/excess: IUGR, PTB, neurodevelopmental alterations	Protective at adequate status/toxic in excess	Excess copper, zinc deficiency
As	Hypertension, hormonal disturbances	IUGR, SGA, congenital anomalies	Toxic	Low methylation capacity; low folate status (± low selenium status)
Pb	Hypertensive disorders, anemia (heme pathway disruption)	Neurodevelopmental outcomes, LBW, PTB	Toxic	Mobilization from maternal bones, tobacco smoke exposure
Cd	Preeclampsia, miscarriage, hormonal disturbances	IUGR, PTB, impaired mineralization	Toxic	Tobacco smoke exposure, low zinc/selenium status

LBW, low birth weight; PTB, preterm birth; IUGR, intrauterine growth restriction; SGA, small for gestational age; GDM, gestational diabetes mellitus.

**Table 3:** Comparative summary of reported associations (direction and qualitative consistency) between individual oncoelements and maternal pregnancy outcomes and fetal/neonatal parameters.

Oncoelements	Maternal					Fetal/Neonatal				Matrix/Timing	Mechanistic Bridge
	PE/PIH	GDM/Glucose	Miscarriage	PROM/pPROM	Preterm	Birth Weight	SGA/IUGR	Length/HC	Apgar		
Se	low → ↑	low → ↑	mixed/ limited	mixed/limited	mixed	low → ↓	low → ↑	limited	limited	maternal blood (early gestation), placenta/cord (mechanistic context)	antioxidant/hyroid/epigenetic [48,49,50,51,52,53]
Zn	low → ↑	mixed	low → ↑ (limited)	mixed	low → ↑	low → ↓	low → ↑	limited	limited	maternal blood; interaction with Cd	DNA repair/immunity/redox [54,55,56,57,58]
Cu	mixed (both low and high → ↑)	high → ↑	low → ↑ (suggested)	mixed	high → ↑	mixed	mixed (low or high → ↑)	limited	limited	maternal blood (10–14 weeks, often reported)	angiogenesis/redox [54,59,60,61,62]
As	high → ↑	high → ↑	high → ↑ (suggested)	limited	high → ↑	high → ↓	high → ↑	high → ↓ (suggested)	high → ↓ (limited)	maternal blood + cord blood	oxidative stress/endocrine/epigenetic [63,64,65]
Pb	mixed	mixed	limited	high → ↑	mixed	high → ↓	high → ↑ (suggested)	high → ↓ (suggested)	limited	maternal blood + cord blood	neurotoxicity/oxidative stress [22,66,67,68]
Cd	high → ↑	mixed	high → ↑	limited	high → ↑	high → ↓	high → ↑	limited	limited	maternal blood (1st/2nd trimester), placenta, cord	oxidative stress/Zn antagonism/epigenetic [14,20,68,69,70,71,72,73]

Arrows indicate the direction of association (*↑* = increased risk or value; *↓* = decreased risk or value). The symbol “→“ denotes “associated with“ and separates the exposure category (‘low’ or ‘high’) from the direction of association (↑/↓) in risk or value. “Low” and “high” refer to relatively lower or higher element levels or exposures, as defined in the original studies. Consistency of findings was visualized using a signal color scheme inspired by the GRADE framework, where green indicated limited evidence, yellow indicated moderate evidence, red indicated high and consistent evidence, and grey denoted mixed or inconsistent findings. The absence of red-coded cells reflected the lack of uniformly high-quality, consistent evidence across studies. Abbreviations: PE, preeclampsia; PIH, pregnancy-induced hypertension; GDM, gestational diabetes mellitus; PROM, premature rupture of membranes; pPROM, preterm premature rupture of membranes; SGA, small for gestational age; IUGR, intrauterine growth restriction; HC, head circumference.

## Data Availability

No new data were created or analyzed in this study. Data sharing is not applicable to this article.

## Abbreviations

AP-1	Activator Protein-1
ATP	Adenosine Triphosphate
ATP7A/B	ATPase Copper-Transporting A/B
BER	Base Excision Repair
CAT	Catalase
DMA	Dimethylarsinic Acid [DMA(V)]
DNA	Deoxyribonucleic Acid
DOHaD	Developmental Origins of Health and Disease
EDCs	Endocrine-Disrupting Chemicals
ER	Estrogen Receptor
GR	Glucocorticoid Receptor
GPx	Glutathione Peroxidase
GSH	Glutathione
hCG	Human Chorionic Gonadotropin
HLA-G	Human Leukocyte Antigen G
hPL	Human Placental Lactogen
HSCs	Hematopoietic Stem Cells
IARC	International Agency for Research on Cancer
IUGR	Intrauterine Growth Restriction
MAPK	Mitogen-Activated Protein Kinases
MMA	Monomethylarsonic Acid
NF-κB	Nuclear Factor kappa-light-chain-enhancer of activated B cells
NMDA	N-Methyl-D-Aspartate Receptor
NER	Nucleotide Excision Repair
Nrf2	Nuclear Factor Erythroid 2-related Factor 2
PlGF	Placental Growth Factor
RNA	Ribonucleic Acid
RNS	Reactive Nitrogen Species
ROS	Reactive Oxygen Species
sFlt-1	Soluble fms-like tyrosine kinase-1
SOD	Superoxide Dismutase
THR	Thyroid Hormone Receptor
TrxR	Thioredoxin Reductase
VEGF	Vascular Endothelial Growth Factor
ZnT1/ZIP8	Zinc Transporter 1/Zrt/Irt-like Protein 8
